# Conserved Microsynteny of *NPR1* with Genes Encoding a Signal Calmodulin-Binding Protein and a CK1-Class Protein Kinase in *Beta vulgaris* and Two Other Eudicots

**DOI:** 10.1155/2008/391259

**Published:** 2008-10-30

**Authors:** David Kuykendall, Jonathan Shao, Tammy Murphy

**Affiliations:** Molecular Plant Pathology Laboratory, Agricultural Research Service, United States Department of Agriculture, 10300 Baltimore Avenue, Building 004, Room 120, Beltsville, MD 20705, USA

## Abstract

*NPR1* is a gene of central importance in enabling plants to resist microbial attack. Therefore, knowledge of nearby genes is important for genome analysis and possibly for improving disease resistance. In this study, systematic DNA sequence analysis, gene annotation, and protein BLASTs were performed to determine genes near the *NPR1* gene in *Beta vulgaris* L., *Medicago truncatula* Gaertn, and *Populus trichocarpa* Torr. & Gray, and to access predicted function. Microsynteny was discovered for *NPR1* with genes *CaMP*, encoding a chloroplast-targeted signal calmodulin-binding protein, and *CK1PK*, a CK1-class protein kinase. Conserved microsynteny of *NPR1*, *CaMP*, and *CK1PK* in three diverse species of eudicots suggests maintenance during evolution by positive selection for close proximity. Perhaps close physical linkage contributes to coordinated expression of these particular genes that may control critically important processes including nuclear events and signal transduction.

## 1. INTRODUCTION

Research done on *Arabidopsis thaliana* (L.) Heynth over a 10-year period in a number of laboratories has amassed considerable evidence that the *NPR1* gene (also called *NIM1*) is of central importance in determining the plant ability to resist microbial attack [1]. In essence, global plant defense responses to pathogen invasion are controlled by the *NPR1* gene product and intracellular redox state, since an inactive dimeric NPR1 protein in the cytosol is reduced to the active monomer which then migrates to the nucleus and activates expression of pathogen-induced “pathogenesis-related” (PR) genes [2].

The central role of NPR1 in positively activating defense mechanisms in response to biotic stress suggests the possibility of enhancing disease resistance in plants by genetic manipulation of the *NPR1* gene. In fact, an increasing number of attempts to improve disease resistance in plants by modifying expression of *NPR1* have been reported [3–7].

Microsynteny is genomics information that can be used to predict the location of homologous genes in different species. Knowledge of microsynteny of genes colinear with *NPR1* in crop species could perhaps be used to devise innovative strategies for molecular genetic modification in order to improve disease resistance. As a step towards identifying genes located near the *NPR1* gene in sugarbeet, a bacterial artificial chromosome (BAC) library [8] was screened and an *NPR1*-carrying clone, SBA091H24, was identified [9]. The *B. vulgaris BvNPR1* gene encodes a predicted protein product being 100% identical to that deduced from the sequence of the cDNA for *B. vulgaris NPR1* (GenBank accession AY640381). SMART analysis of the predicted *BvNPR1* gene product [9] showed a BTB/POZ domain and two ARDs, or ankyrin repeat domains [10], both being characteristic of NPR1 proteins and other transcriptional activators within the nucleus. *NPR1* is responsible for disease resistance priming or “induced resistance,” a result of coordinated expression of multiple defense mechanisms/pathways to effectively resist microbial attack [11].

The *NPR1* gene in sugarbeet has been only recently shown [12] to be essential for induced systemic resistance, as in the model *A. thaliana* (L.) Heynth [1]. In both plant species, the activated form of NPR1 migrates into the nucleus and activates the transcription of genes involved with resisting disease-forming microbes [12].

Conservation of microsynteny among distinct families of eudicots was discovered in *Lycopersicon esculentum* Mill. (tomato) and *A. thaliana,* where large-scale duplications followed by selective gene loss have created a network of chromosomal synteny [13]—an accepted paradigm. By developing physical genetic maps based on expressed sequence tags (ESTs), Dominguez et al. [14] discovered conserved synteny with *Arabidopsis* among the genomes of four phylogenetically divergent eudicot crops, namely, sugarbeet, potato, sunflower, and plum.

In this study, complete BAC sequence analysis identified two core plant genes tightly physically linked to *NPR1*, and established a conservation of microsynteny between the *NPR1* gene regions of sugarbeet and two other eudicot species. We report the gene content and organization of a 130 Kb DNA contig (continuous fragment) from an *NPR1*-carrying sugarbeet BAC. Comparison of similar *NPR1*-carrying DNA contigs from *M. truncatula* and *P. trichocarpa* showed that orthologs of genes encoding NPR1, a signal-peptide calmodulin-binding protein (CaMP) and a CK1-class dual-specificity protein kinase (CK1PK) occur in the same order and with a conserved direction of transcription in three divergent species of eudicots. This suggests positive natural selection for maintaining the physical proximity of genes whose products control certain essential nuclear events and a particular signal transduction function, as yet undefined.

## 2. MATERIALS AND METHODS

### 2.1. DNA sequencing

Genomic DNA of *B. vulgaris* hybrid US H20 [15], with an estimated 750 Mb genome size, had previously been used to construct a BAC library by ligating large DNA fragments resulting from partial *HindIII* digestion into vector pECBAC1 [16]. About 34,500 clones comprised the BAC library, and the average insert size was about 120 Kb, providing about 6.1X genome coverage [8]. Primers designed on the basis of data extracted from GenBank accession AY640381, a cDNA sequence for *B. vulgaris NPR1*, were utilized to screen and identify a *BvNPR1*-carrying BAC [9]. The presence of a complete genomic *BvNPR1* gene was established by partial DNA sequence analysis of BAC clone SBA091H24 (GenBank accession DQ851167) [9].

BAC sequencing was completed at Washington University's Genome Sequencing Center in St. Louis, Mo, USA (http://genome.wustl.edu/). The BAC clone SBA091H24 was provided to the Genome Sequencing Center as a glycerol stock. Purification, library construction, shotgun cloning, and sequence analysis were performed on a sufficient number of random subclones to provide about 9.4X coverage. ABI 3730 capillary sequencers were used. Data was assembled using the phred/phrap suite (http://www.phrap.org/).

### 2.2. Gene annotation

Analysis of the sequence data was performed using Lasergene (DNASTAR, Inc., Madison, WI, USA) for assembly and NCBI BLAST [17]. The sequence contig was screened for coding sequence using a combination of the following programs: GeneMark [18, 19] for eukaryotes (http://exon.gatech.edu/GeneMark/eukhmm.cgi), GenScan (http://genes.mit.edu/GENSCAN.html), FGENESH (http://softberry.com/), and GRAIL (http://grail.lsd.ornl.gov/grailexp/). In all cases, *A. thaliana* was chosen as a model, and default settings were used. BLASTP searches were performed at the National Center for Biotechnology Information (NCBI) website (http://www.ncbi.nlm.nih.gov/BLAST/). Percent identities and percent similarities were obtained using BLAST alignments. Manual curation of proteins was performed using Lasergene MegAlign and EditSeq sequence analysis software, where applicable simple modular architecture research tool (SMART) [20] database (http://smart.emblheidelberg.de/) and Motif Scan (http://myhits.isb-sib.ch/cgi-bin/motif_scan) were used to identify protein domains and motifs, respectively. ARTEMIS (http://www.sanger.ac.uk/Software/Artemis/) was used to collate data and facilitate annotation.

Calmodulin target database (http://calcium.uhnres.utor-onto.ca/ctdb/ctdb/home.html) was used to identify the most likely calmodulin-binding site in a peptide sequence. The hmmtop server (http://www.enzim.hu/hmmtop/) was used to predict transmembrane helices, and a signal peptide was detected using the SignalP 3.0 and TargetP 1.0 servers (http://www.cbs.dtu.dk/services/).

BAC chromosome and genomic annotation datasets for *M. truncatula* and *O. sativa* L. were accessed through TIGR (http://www.tigr.org/). *P. trichocarpa* (black cottonwood) genome information [21] was accessed through NCBI (http://www.ncbi.nlm.nih.gov/entrez/query.fcgi?db=genomeprj&cmd=Retrieve&dopt=Overview&list_uids=10770).

### 2.3. Identification of colinearity

BLAST searches were performed for protein products of the predicted ORFs of the *B. vulgaris NPR1* BAC against databases for *A. thaliana, M. truncatula, P. trichocarpa,* and *O. sativa* L. ssp. *japonica*. High-scoring pairs (HSPs), the predicted protein products with highly significant matches, were considered as products of orthologous genes. Corresponding DNA regions are considered to be microcolinear when two or more orthologous genes are present in physical proximity, in the same order, and are transcribed in the same direction.

GenBank accession NC_008472.1 is representative of linkage group VI of *P. trichocarpa* [21], and the subsets of particular interest were coordinates from 8404895 to 8453314. GenBank accession AC124609 represents BAC clone mth229b13, a subset of chromosome 2 of *M. truncatula* (http://www.tigr.org/tigr-scripts/medicago/contig_location_association.pl?chromosome=2) [22, 23]. AC124609 (1...61740 bases) was extracted and compared to our *B. vulgaris* GenBank accession EF101866 (1...129695 bases).

## 3. RESULTS

**Figure 1:**
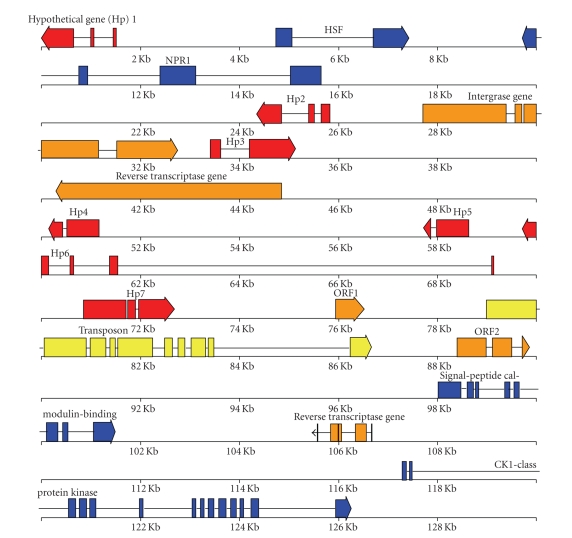
Schematic representation of genes annotated on the 130 Kb genomic *NPR1*-carrying BAC from sugarbeet (GenBank accession EF101866) based on FGENESH, GenScan, GeneMark, and GRAIL gene finders as well as SMART and Motif Peptide Scan results. (Blue: core plant genes involved with nuclear events/signal transduction/defense; yellow: transposon; orange: retrotransposons; red: encode only hypothetical or unknown proteins). The predicted genes begin with a bar and end with an arrowhead, thus indicating the direction of transcription.

A 130 Kb BAC designated SBA091H24, containing *B. vulgaris* chromosomal DNA, more specifically the sugarbeet *NPR1* gene [9], was sequenced and fully annotated (GenBank accession EF101866). The bioinformatics tools FGENESH, GeneMark, GenScan, and GRAIL were used as gene finders. Predicted gene names and predicted functions of deduced amino acid sequences, where possible, are presented in Table 1, and a visual representation of exon structure is shown in Figure 1. On the 130 Kb contig, a total of 17 potential open reading frames (ORFs), or protein coding regions, were identified. Only four out of the 17 open reading frames (ORFs) were predicted to produce protein products with high amino acid similarity to known products of core plant genes (Table 1). In addition to four core plant genes, the 130 Kb contig contains five retrotransposon genes, a transposon gene, and seven other genes whose products lacked a predicted function.

In addition to *NPR1*, another core plant gene, predicted on the 130 Kb sugarbeet DNA contig from BAC clone SBA091H24, was composed of two exons that encode a heat shock factor (HSF) protein with a conserved DNA binding domain (*E* = 2e^−29^) from amino acid positions 45 to 205. This *HSF* gene is located between the *NPR1* gene and gene *Hp1*, encoding a hypothetical protein with some similarity to retrotransposon-encoded proteins. The *HSF* gene and *NPR1* are transcribed in opposing directions. The predicted HSF protein has moderately high similarity (Table 1) to the protein product of *HSFA9*—a leaf pattern morphogenesis-controlling gene of sunflower, *Helianthus annuus* [24].

Beginning at about 70 Kb upstream of *NPR1*, another core plant gene encodes a calmodulin-binding protein (CaMP) that, as SMART analysis revealed, has an IQ domain from amino acid positions 131 to 153. Motif Scan suggested (1) an IMP dehydrogenase/GMP reductase domain at amino acids 121–444, (2) several protein kinase phosphorylation sites, and (3) an involucrin repeat at amino acids 206–215. The predicted protein product gave numerous BLAST hits to calmodulin-binding proteins. Calmodulin target database predicted amino acid positions 201–209 as the most likely calmodulin-binding site. Transmembrane helices were predicted at amino acid positions 337–353 by the hmmtop server, and the SignalP 3.0 server predicted a cleavage site in the N-terminal signal-peptide sequence between glutamine and lysine in amino acid positions 17 and 18. Thus, we deduced that the mature CaMP protein is 508 amino acids in length. The pro-CaMP was predicted as 525 amino acids in length, and the presence of a membrane-targeted N-terminal signal-peptide sequence suggests that peptide maturation occurs upon passage into or through a membrane. TargetP predicted that the targeted sites of subcellular localization of mature protein were chloroplasts.

Beginning at about 90 Kb upstream of *NPR1*, a third core plant gene encodes a “casein kinase class 1” protein kinase (CK1PK), identified by numerous BLAST hits with *E*-values of 0 to CK1PKs. SMART analysis revealed a dual-specificity STYKc protein kinase domain from amino acids 9–211. This type of kinase phosphorylates either serine, threonine, or tyrosine residues.

**Figure 2:**
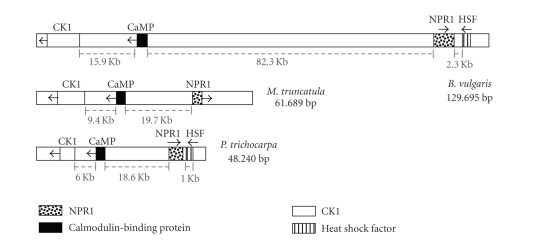
Schematic representation of the microcolinearity found between *NPR1*-carrying regions of (a) *B. vulgaris* (GenBank EF101866), (b) *M. truncatula* (GenBank AC124609.20), and (c) a subset of *P. trichocarpa* (NC_008472.1). Genes are indicated by boxes: *NPR1* (gray box); *CaMP* encoding a calmodulin-binding protein (black box); specifying a *CK1PK*-class protein kinase (white box). Arrows indicate the direction of transcription. The gray bars indicating the distances in Kb between genes are from the end exon of one gene to the end exon of the next gene. The length of the DNA regions under comparison is indicated in base pairs. As of this writing, the TIGR annotated *NPR1* region for *M. truncatula* is a draft; shown is only about one half of a BAC sequence since there was a gap in the sequence near the center.

Conservation of microsynteny was discovered in *B. vulgaris*, *M. truncatula*, and *P. trichocarpa* (Figure 2), but not in *A. thaliana* or *O. sativa L.*, by comparative DNA analysis of three core plant genes: (1) a CK1 class dual-specificity protein kinase gene, (2) a signal calmodulin-binding protein gene, and (3) the disease resistance-priming *NPR1* gene. The high degree of amino acid similarity as well as identity of the predicted products of these respective microcolinear genes (Table 2) clearly indicates that they are orthologous gene pairs with shared functions.

The three orthologous gene pairs *NPR1, CaMP,* and *CK1PK* are colinear, that is, with both the same gene order and direction of transcription, in *B. vulgaris*, *M. truncatula,* and *P. trichocarpa* (Figure 2). In particular, in the comparison of presumptive orthologous gene pairs in *B. vulgaris* and *M. truncatula,* the *NPR1, CaMP,* and *CK1PK* genes encode proteins that have amino acid identities of about 60%, 57%, and 67%, and that exhibit amino acid similarities of about 74%, 68%, and 77%, respectively (Table 2). Similarly, in the comparison of presumptive orthologous gene pairs in *B. vulgaris* and *P. trichocarpa,* the three orthologous gene pairs, (*NPR1, CaMP,* and *CK1PK*), produce protein products with amino acid identities of about 65%, 56%, and 75% and amino acid similarities of about 79%, 68%, and 82%, respectively (Table 2). Finally, comparison of the amino acid identities and similarities of the predicted products of the *NPR1*, *CaMP,* and *CK1PK* genes in *P. trichocarpa* and *M. truncatula* (Table 2) indicates conserved orthologous gene pairs.

As mentioned above, about 2 Kb downstream of *NPR1* in *B. vulgaris,* a fourth core plant gene (Figure 2) was predicted to produce a protein product with moderately high similarity to that produced by the embryonically expressed heat shock factor (HSF) *A9* gene in sunflower. Microcolinearity of this particular *HSF* gene with *NPR1* did not occur in *M. truncatula*. On the other hand, *NPR1* in *P. trichocarpa* is separated from an *HSF* gene by only 1 Kb, but it encodes a protein that has only about 39% amino acid identity and 54% amino acid similarity with the protein encoded by the *HSF* gene adjacent to the *NPR1* gene in *B. vulgaris*. Thus, the structure and function of the respective HSF proteins is not as highly conserved as were the three other colinear core plant genes in poplar and sugarbeet.

Comparison of the *Arabidopsis NPR1* region with those of *M. truncatula*, *P. trichocarpa,* and *B. vulgaris* revealed that *Arabidopsis* lacks conserved microsynteny of *CaMP* and *CK1PK* genes with *NPR1*. In *O. sativa L.*, the *NPR1* genomic region has a gene encoding a calmodulin-binding protein, but it is transcribed in the same direction as *NPR1* rather than in opposing directions as in *B. vulgaris* and *M. truncatula*; also the *CK1PK* gene, most proximal to *NPR1* in *O. sativa L.*, is greater than 250 Kb away (not shown). Thus, microsynteny, as in three out of four eudicot species, did not occur in *O. sativa L.*, perhaps not unexpectedly as it is a monocot.

The six other noncore genes were retrotransposon or DNA transposon ones. Four putative genes were predicted to encode proteins with strong BLAST hits to retrotransposons or retrotransposon-like genes. An integrase gene has four exons and, from amino acid positions 750 to 900, this ORF encodes an *rve* core domain (*E* = 3e^−30^), being a characteristic of integrases. With the integrase gene and the downstream putative gene *Hp3* being combined, both share about 98% nucleotide identity (BLASTN) with highly related genes on a 9 Kb DNA contig (GenBank accession ABD83280) of BAC62 from sugarbeet chromosome 9 [26]. A putative reverse transcriptase gene about 5 Kb downstream of *Hp3* consists of a single exon encoding a polyprotein with *Rvt2* RNA-dependent DNA polymerase domain (*E* = 7e^−24^), an *rve* integrase domain (*E* = 6e^−21^), and a poorly conserved (*E* = 8e^−10^) *gag* capsid-like protein domain.

*Coe1*, previously identified by our group using LTR_STRUC analysis as a novel composite of class I and class II elements [25], has three genes. Briefly, one gene has a single exon ORF1 (Table 1), encoding a retroelement-like protein. ORF1 is about 2 Kb upstream of a gene that encodes a DNA transposase since its predicted product produced many significant BLAST alignments with DNA transposases, with *E*-values equal to 0. The very highly conserved transposase family *tnp2* domain (*E* = 1e^−94^) occurs in amino acid residue positions 200–400 of the predicted protein. *Coe1*'s transposase gene is also flanked by ORF2, a pseudogene of a *copia*-like integrase, and all three genes are within LTRs [7]. ORF2 is 2 Kb downstream of the *tnp2*-class DNA transposase gene, and its predicted product showed, by BLAST analysis, a significant alignment with several putative retrotransposon proteins from wine grape and rice; for example, it aligned to *O.* sativa's putative *Ty1-copia* subclass retrotransposon (accession ABA95677) with *E*-value of ≤ e^−50^, 38% amino acid identity, and 49% amino acid similarity.

Downstream of the gene encoding a calmodulin-binding protein, a putative reverse transcriptase gene encoded a predicted protein with only a moderate BLAST alignment (*E* = 3e^−37^) to a reverse transcriptase *Rvt-2* domain found in a novel retrotransposon-like TIR-NBS-LRR-type disease resistance protein in *P. trichocarpa* Torr. & Gray (Table 1), and the similarity corresponds to a shared *Rvt-2*-type domain.

In *M. truncatula,* a TIR-NBS-LRR-type resistance gene occurs downstream of *NPR1* (not shown). The predicted product of the *M. truncatula* resistance gene analogue (RGA) near *NPR1* has 39% amino acid identity and 59% amino acid similarity with the *Gro1-4* nematode resistance gene of *Solanum tuberosum* (GenBank accession AY196152.1).

Comparative sequence analysis and gene annotation in *B. vulgaris, M. truncatula,* and *P. trichocarpa* revealed that these three divergent species of eudicots exhibit conserved microsynteny of genes that encode the centrally important disease resistance priming NPR1, a signal-peptide calmodulin-binding protein (CaMP), and a CK1-class dual-specificity protein kinase (CK1PK). The orthologs occur in the same order and with the same direction of transcription.

## 4. DISCUSSION

In this study, comparison of orthologous *NPR1* gene regions of *B. vulgaris*, *M. truncatula,* and *P. trichocarpa* revealed for the first time conserved microsynteny of the defense-priming *NPR1* gene with a *CaMP* gene, encoding a calmodulin-binding protein, and with a *CK1PK* gene, specifying a CK1-class dual-specificity protein kinase.

Calmodulin-binding proteins in plants are very diverse, exhibit various motifs, and perform a correspondingly wide variety of functions [27–30]. For example, an *Arabidopsis* ethylene-upregulated calmodulin-binding protein triggers senescence and death [31].

Calcium and calmodulin mediate a complex signal transduction network in plants through protein kinases (PKs), and some PKs are unique to plants [32]. They are literal “hubs” of sensory perception and signal transduction. Examples include a calmodulin-binding PK in *Arabidopsis* that negatively regulates tolerance to osmotic stress [32] and a calmodulin-binding PK in tobacco (*Nicotiana tabacum* L.), involved with negative regulation of flowering [33].

It seems reasonable to hypothesize that the *CaMP* gene product, which is a chloroplast-targeted, signal-peptide, calmodulin-binding transmembrane protein, could play a role in rapid activation of a defense cascade during either general stress or pathogen response. Just as the *NPR1* gene is critical for disease resistance priming in plants, some calmodulin-binding proteins are pathogenesis-related. For example, de novo synthesis of a calmodulin-binding peptide with a DNA-binding domain at the N-terminus is induced by ethylene formed by the plant in response to wounding and/or infection [34]; also in *Arabidopsis*, another calmodulin-binding protein is pathogenesis-related as well [35]. Moreover, in *Glycine max* L. (soybean), specific calmodulin isoforms are required for the expression of pathogen-induced proteins upregulated by the *NPR1* disease resistance control gene [36]. Conserved microsynteny of *CK1PK, CaMP,* and *NPR1* genes, discovered in three out of four eudicot species examined, could be hypothesized to suggest that their protein products play essential cellular roles related to plant defense response.

We propose a new hypothesis that conserved gene microsynteny of certain core plant genes in eudicots may correlate with either similar subcellular localization or with similar function. Either possibility for the protein products of the three core plant genes herein described is plausible. Activated monomeric NPR1 functions in the plant nucleus, where CK1PKs are also localized. CK1s are believed to control circadian rhythm [37] and chromosome partitioning during meiotic cell division [38] in all eukarotic cells. It should be noted that in *O. sativa* a novel family of dual-specificity PKs is involved in controlling the plant responses to biotic as well as abiotic stresses [39]. Based on available literature [30, 31, 39], the *CK1PK* gene localized near *NPR1* in certain eudicots may play a role in controlling the expression of stress-responsive genes in plants.

A total of 11 retrotransposons (RTs), DNA transposons, and hypothetical genes lie within the approximately 80 Kb stretch of DNA between the *NPR1* and *CaMP* genes in *B. vulgaris*; therefore, we conclude that this region is rich in repetitive elements, and several insertions of mobile genetic elements have occurred during evolutionary divergence (Kuykendall et al., unpublished). ORFs, originating from either retrotransposons or viruses, from DNA transposons and other repetitive elements need not be considered disruptive of the conservation of colinearity when the core genes nevertheless occur in physical proximity, in the same order, and are transcribed in the same relative direction. Fortunately, our approach was not to be dissuaded by the repetitive elements that occur between *NPR1* and *CaMP* in *B. vulgaris* comparing the orthologous regions of *M. truncatula* and *P. trichocarpa,* and thus we discovered conserved microsynteny for orthologous *NPR1, CaMP,* and *CK1PK* genes in these three eudicot species. This conserved microsynteny of *NPR1, CaMP,* and *CK1PK* in *B. vulgaris*, *M. truncatula,* and *P. trichocarpa* likely has an evolutionary basis through a yet undefined selective advantage.

The observed conservation of microsynteny of the three core plant genes could be hypothesized to result, in part, from positive natural selection for physical proximity. Hypothetically, close physical linkage may facilitate coordinated expression of genes critical in certain controlling nuclear events, such as those for which the protein products of *CK1PK* and *NPR1* are known, or are responsible, either directly or indirectly, for the initiation of a dynamic signal transduction cascade as can be hypothesized for the predicted product of *CaMP*.

Reverse transcriptase (RT) PCR expression studies will be useful in determining whether the *CaMP* or *CK1PK* is upregulated in response to the administration of factors which induce the production of either pathogenesis-related or stress-related proteins. Such studies will hopefully allow one to determine whether the chloroplast-targeted signal-peptide calmodulin-binding protein gene (*CaMP*) or the nuclearly localized casein kinase 1 protein kinase gene (*CK1PK*) may play a role(s) in pathogen and/or stress response.

In summary, in addition to *NPR1* gene and the *CaMP* and *CK1PK* genes herein described for the first time, the 130 Kb *NPR1*-carrying *B. vulgaris* genomic DNA segment has 14 other features predicted by gene finders trained with *Arabidopsis*. Whereas seven ORFs produce predicted proteins with probable functions that can be deduced from BLAST analysis, seven other ORFs had predicted protein products without any known function.

It is also interesting to note that an *HSF* gene is located just 2 Kb downstream of *NPR1* in sugarbeet, and the predicted product of this gene is a DNA-binding HSF protein similar to that specified by the *HSFA9* gene that controls early leaf morphogenesis in sunflower [24].

We conclude that conserved microsynteny of *NPR1, CaMP,* and *CK1PK* in three eudicot species suggests strong positive natural selection for the maintenance of physical linkage of these particular genes whose vital protein products either control specific nuclear events or are involved in signal transduction.

## Figures and Tables

**Table 1:** Predicted genes and their designated functions.

Gene	Product length (a.a.)	Best BLAST Hit^a^	*E*-value	Similarity	Designation^b^
Hp1	259	ABE85118	5.0*E*−08	49/101	Hypothetical^c^

HSF (similar to HSFA9)	337	AAM43804	3.4*E*−47	185/320	HSF transcription factor

NPR1	604	AAT57640	0.0*E*+00	604/604	NPR1 disease resistance

Hp2	259	none			Hypothetical^c^

Integrase	1516	ABE91091	0.0*E*+00	903/1535	Integrase (*copia* type)

Hp3	403	ABD83280	1.1*E*−124	315/325	Unknown^d^

Reverse transcriptase (RT)	1501	ABE83303	0.0*E*+00	996/1503	*Gypsy*-type retrotransposon

Hp4	302	CAH67120	1.0*E*−13	78/177	Hypothetical^c^

Hp5	263	ABD83301	1.0*E*−09	41/63	Hypothetical^c^

Hp6	222	none			Hypothetical^c^

Hp7	593	ABD83280	4.1*E*−60	186/282	Unknown^d^

ORF1 of *Coe1* (a composite of class I and class 2 elements) [25]	188	NP_199616.1	1e−17	106/192	Hypothetical (retroelement-like gene)

*Coe1* (transposon gene)	1399	ABE82848	0.0*E*−00	604/956	Transposon

ORF2 of *Coe1* (an integrase pseudogene)	297	ABA95677	4.0*E*−70	177/298	*Rvt2* domain integrase pseudogene

Calmodulin-binding protein (CaMP)	525	NP_974673	7.0*E*−161	361/495	Signal-peptide calmodulin-binding protein

RT (reverse transcriptase)	198	ABF81417	3.0*E*−37	126/213	RT-like gene

CK1-class protein kinase (CK1PK)	473	BAB92346	0.0*E*+00	411/476	CK1-class dual-specificity protein kinase

^a^GenBank accession number or protein ID of the best BLAST hit, followed by the *E*-value and percent (similar/total amino acids) similarity between the query and the best hit. ^b^Designation based on the deductions possible via bioinformatics tools listed in Section 2. Functional classification based on the result of protein BLAST search. ^c^NA: not applicable; putative function of the product was not identified. ^d^Similar to FGENESH 21 [26].

**Table 2:** Percent amino acid identity and similarity exhibited by amino acid alignments of the conserved products of orthologous gene pairs.

Orthologous	*B. vulgaris* NPR1	*B. vulgaris* CaMP	*B. vulgaris* CK1PK
gene pair	Identity/similarity	Identity/similarity	Identity/similarity
*Medicago* homologs	60%/74%	57%/68%	67%/77%
*Populus* homologs	65%/79%	56%/68%	75%/82%

	*Medicago* NPR1	*Medicago* CaMP	*Medicago* CK1PK
	Identity/similarity	Identity/similarity	Identity/similarity

*Populus* homologs	62%/75%	60%/71%	69%/78%
